# The Impact of COVID-19 on Palliative Care: Perspective of Healthcare Professionals

**DOI:** 10.7759/cureus.19522

**Published:** 2021-11-13

**Authors:** Pedro Tavares, Carlos Rodrigues, Isabel G Neto

**Affiliations:** 1 Palliative Care Department, Hospital da Luz Lisboa, Lisboa, PRT

**Keywords:** coronavirus, pandemic, palliative care, impact on care, qualitative study

## Abstract

The COVID-19 pandemic has negatively impacted the provision of Palliative Care (PC), challenging the teams aiming to provide adequate care.

This is a qualitative study that intends to know, from the perspective of health professionals of a Palliative Care Unit (PCU), the main challenges in providing PC during a pandemic and describes the strategies to be adopted to solve the identified difficulties. We utilized the content analysis, according to Bardin, for data analysis of written narratives of health professionals from a PCU (n=14). In the analysis of the difficulties in providing PC, from the perspective of health professionals, were identified five themes: 1) Altered relationship between healthcare professional and patients, 2) altered relationship between healthcare professional and family, 3) altered communication with patients and their family, 4) altered working dynamic within the PCU, and 5) altered use of healthcare resources. Concerning the strategies to be implemented, the health professionals identified two themes: 1) Strategies to implement between the patient and family members and 2) strategies to implement for the healthcare professionals.

The provision of PC is affected by changes in the relationship between the health professional and the patient/caregiver or family, in communication with the patient/caregiver or family, and the use of health resources. In an attempt to lessen the impact of these changes, participants identified strategies to improve PC delivery in these circumstances. During this pandemic, it is imperative to implement rigorous strategies for managing specialized human resources. Physical distance and personal protective equipment are barriers to communication and emotional support, which is essential in PC and this barrier is further accentuated by the required physical distance from family members and caregivers. PC´s main aim continues to be the mitigation of suffering.

## Introduction

Since the beginning of 2020, healthcare, in general, and also Palliative Care (PC), have undergone important changes. On March 11, 2020, the infection caused by the new SARS-CoV-2 coronavirus became an international pandemic [1]. Over the months that ensued, as the number of infected people increased, the COVID-19 pandemic caused an increase in the number of hospital admissions, affected health systems, and had implications for access to healthcare, with a significant reduction in appointments, diagnostic tests, and surgical interventions, and Portugal was no exception [2]. The increase in the number of deaths, directly and indirectly, related to COVID-19, implied changes in healthcare provision. In Portugal, between March 2, 2020, when the first cases of COVID-19 were diagnosed, and 30 August 2020 there were 57,971 deaths, an increase of 6,312 deaths compared to the same time periods of the last five years. Of the total number of deaths, only 1,822 were as a result of SARS-CoV-2 infection [3].

This increase in mortality significantly transformed the functioning of the healthcare system and the attempt to minimize damage altered the face of PC provision. To prevent the disease itself, there was an immediate and rigorous response from health services, creating different circuits for patients with suspicion of COVID-19 infection and significantly reduced face-to-face care, where only acute situations or exacerbations of chronic illnesses were attended. On the other hand, due to the measures adopted by the competent authorities with a view to reducing the transmission of the disease, restrictions were used, in particular circulation rights which included limiting inpatient visits [4].

The impact of COVID-19 reached all levels of healthcare and PC was no exception [5]. Modifications were implemented, seeking to guarantee protection for patients, healthcare professionals, and family members, in order to achieve a greater objective: the control of the pandemic. The pandemic delayed and, in some cases, reversed advances achieved so far in terms of PC development [6]. Access to PC has changed, along with changes for all areas of the health sector. However, the PC response, although still insufficient is emerging in the face of this pandemic [7].

PC is a clinical area of ​​specialization, as is the case with so many other medical and nursing specialties. PC's main target is the active intervention in the prevention and relief of suffering resulting from serious, life-limiting, or irreversible diseases [8]. PC promotes human dignity, which is also affected by COVID-19, and seeks to improve the quality of life (QoL) of patients, family members, and caregivers [9,10]. Even before the pandemic, access to PC was limited worldwide; in Portugal, only 25% of adults and 0.01% of pediatric patients had access to PC [6,11,12].

The role of PC in this pandemic is of recognized importance [4]. Their role is to support PC patients who became infected, patients with SARS-CoV-2 who have palliative needs, and in the care and treatment of non-COVID patients with palliative needs [13,14]. The intervention by the PC teams takes place in all aspects of suffering, in symptomatic control and psychosocial support, in making complex decisions, and in the anticipated discussion of patient’s wishes [14,15].

The correct management of health resources may imply ethical dilemmas, based on the principles of justice and beneficence, such as when deciding who should or should not receive or have access to intensive care interventions, withholding certain medical interventions. It is important that, above all, all patients receive care that promotes their dignity [4]. For those whose prognosis may be limited, it is essential that they receive high-quality PC, as an essential part of their healthcare plan [6]. The relevance of intervention in suffering should not conflict with the preservation of life [16]. In our Palliative Care Unit (PCU), during the pandemic, we sought to harmonize the limitation of visits and social distancing, with end-of-life situations, allowing exceptional visits with the appropriate protection measures, combined with the use of technology, allowing communication at a distance.

Considering the public health emergency that occurred and the exceptional situation that was experienced and currently persists, it is important to assess its impact on the provision of PC. This study explores, from the perspective of the health professionals of a PCU, the difficulties in providing care during a pandemic and which strategies to implement to solve the identified difficulties.

## Materials and methods

Study design

The qualitative study focused on the analysis of data collected from health professionals in a PCU.

Study population

Health professionals’ convenience sample at the Hospital da Luz Lisboa PCU, Portugal. We include health professionals who had basic training in PC (between 18 and 45 hours of training). Exclusion criteria included health professionals with less than one year of professional experience in PC, professionals with prolonged absences, equal to or over 30 days from the declaration of the pandemic by the World Health Organization and professionals who, since the date of the declaration of the pandemic, have performed functions, regardless of its duration, in services dedicated exclusively to COVID-19. The invitation to participate was sent on June 25, 2020, a reminder on July 14, and the questionnaire ended on August 12, 2020. 

Data collection

The data was collected through a written questionnaire sent by email, with open questions, with no word limit. In addition to aspects that allowed for a sociodemographic characterization, the questionnaire included three open-answer questions:

- What are, in your perspective, the added difficulties experienced in providing palliative care, considering the current epidemiological context?

- Considering the difficulties identified, what strategies could be implemented to resolve these difficulties?

- In your opinion, what conclusions should be drawn from the current epidemiological context that can be implemented in a post-pandemic context?

Data analysis

The data analysis was based on the method of content analysis by Bardin (2011) [17], using the ATLAS.ti® software as an auxiliary tool in data organization. 

For data presentation, each participant was identified with the letter “I,” followed by a number in ascending order, starting with one, according to chronological acceptance of participation in the study. In the results presentation, we use linguistic symbols/codes, whose meaning we explain:

- “Phrases in quotation marks” - Correspond to interviewees' quotes (words or phrases);

- (...) - Quotes omission (considered not relevant for analysis).

Due to the nature of the study and the data, no statistical treatment was performed.

Ethics

This study was authorized by the research committee of the Hospital da Luz Lisboa and approved by the Ethics Committee for Health of Hospital da Luz (Approval no. CES/09/2021/ME). The collection of information was carried out anonymously, guaranteeing respect for Human Rights and professional ethics. The participants received detailed information and their participation was voluntary, free, and written informed consent was obtained. Data collected was only used as a part of this study. This study did not present harm, benefits or costs for the research subjects.

## Results

The sample consists of a total of 14 participants, including eight nurses, three doctors, one psychologist, one pharmacist, and one physiotherapist, and includes individuals between 26 and 50 years old, with an average age of 35.4 years. As for gender, the sample is predominantly female, 85.7% (n=12), and 14.3% (n=2) male.

In terms of professional practice, the sample has professional experience between five and 25 years of practice, with an average of 12.1 years. As for professional practice in PC, the average is six and a half years of professional practice, ranging between four and twelve years.

Regarding academic training in PC, three of the participants have basic training, nine have a postgraduate degree in PC, and two have a master's degree in PC. From the total of 14 participants, one nurse is a specialist of nursing for people with palliative needs and one doctor has recognized competence in palliative medicine.

The tables below reflect the obtained results: the increased difficulties in providing PC (Table 1) and the strategies to resolve the difficulties identified by health professionals (Table 2).

**Table 1:** Categories and sub-categories: added difficulties in providing PC from the perspective of health professionals.

Theme	Category	Sub-category	Frequency
Altered relationship between healthcare professional and patients	Limitations to the use of dignity promoting strategies		7
Altered relationship between healthcare professional and family	Limited visits	Risk of complicated grief	4
		Compromised family support	5
Altered communication with patients and their family	Need to share information over the phone	Physical barriers; use of telephone	9
	Use of personal protective equipment and altered verbal and non-verbal communication	Altered non-verbal communication such as touch	8
		Perception of distance	11
		Perceived abandonment	6
Altered working dynamic within the PCU	Admitting patients with palliative needs to the respiratory circuit	Increased time to symptomatic control	9
		Lack of adequate procedures and protocols	7
	Relocation of human resources to “COVID wards” with consequent altered PC team	Reduction in PC team numbers	9
		Health professionals with stress and anxiety- Risk of Burnout	7
	Compromised data collection and patient observation	Compromised symptom control	8
Altered use of healthcare resources	Fear of accessing healthcare	Late diagnoses	6
		Late referral to PC	4
		Compromised symptom control	9

**Table 2:** Overview of categories and subcategories: strategies to be implemented from the perspective of health professionals.

Theme	Category	Sub-category	Frequency
Strategies to implement between the patient and family members	Strategies that promote effective communication	Programmed telephone contact, promoted by healthcare professionals	9
		Early establishment of who is the family member or person of reference for the transmission of information related to the hospitalized patient and decision making	7
		Family conference after admission	7
		Promote telephone appointments	10
		Promote training in communication skills	8
	Strategies to help with negative emotions felt by patients and their families	Allow a family member to be present full time	5
		Allow post mortemvisits	4
		Promote video calls between patients and their family members	7
		Promote the use of digital platforms to augment patient inclusion and socialization	7
	Definition of visits		8
Strategies to implement for the healthcare professionals	Strategies that promote well being		6
	Create protocols and procedures adapted to the pandemic		7
	Improve communication and organization in the emergency department		6

## Discussion

Added difficulties in providing PC from the perspective of health professionals

Public health restrictions, imposed by the pandemic, contribute to patients dying alone [6]. 

“It is difficult for the patient who finds himself unable to be with the people he loves, often at the end of his life, and for families in which the feeling of helplessness and guilt - a sense of abandonment - will forever have repercussions on the grieving process.” I8

For all who are in the last days and hours of life, despite the pandemic, protecting human dignity is paramount [4,18]. The hospitalized patient when in isolation, is more likely to experience depression and anxiety, along with a decrease in self-esteem. The reduction in the time that health professionals spend with patients contributes to this. There is also anxiety and fear of health professionals who realize the impact of their presence on the safety of the patient as well as their own [6,19].

Furthermore, the lack of social and family support, not feeling valued or respected, can reduce the person's perception of dignity [18].

“In promoting dignity, all actions, often religious and even the fulfillment of last wishes, are completely suspended during the pandemic.” I8

During this pandemic, people who find themselves isolated experience additional suffering [20]. Circulation limitations, combined with restrictions on visits by family members, negatively impact the patient [4].

According to Byock [16], there are five major tasks at the end of life: telling people you care about how you feel, forgive, be forgiven, say thank you, and say goodbye. The pace at which people live can lead them not to do this throughout their lives and, at the end of their lives, it will be an experience of personal re-encounter, which can be gratifying not only for themselves. These tasks have been affected by the pandemic, which can, as the participants of this study report, contribute to putting families at risk of complicated and even pathological grief [10,21,22].

“The outcome of the disease situation culminates, as is often expected, in death and in this pandemic, it is disconcerting for family members and loved ones not being able to participate, as usual, in the last moments of life. Something that, for those who lose someone, is very pacifying, there is a moment of farewell, so that the mourning process can be lived with ‘normality’.” I4

Although not reported by the participants, we witnessed the postponement of mourning rituals, prioritizing the fight against the public health threat, to the detriment, for example, of funeral rituals. Farewell rituals promote quality of life for family members and can contribute favorably to the resolution of grief [23].

The pandemic forced new routines to be established. Participants report that one of the difficulties in providing PC is the increased use of the telephone.

The pandemic challenged PC health professionals to one of their fundamental commitments, non-abandonment [5].

Although the use of telephones is reported by most participants as a strategy to reduce the feeling of abandonment, both by patients, but also by family members, participants report that, combined with visit restrictions, the use of the telephone makes it difficult to provide care, as it implies additional time and there is not always guidance on where, who, and when contact is made. There is also the detriment of face-to-face contact which also affects the care provided. The family and significant others are the targets of care, and, during the pandemic, the limitation of their presence negatively impacts the patient [5].

“Limitation of visits to family members and significant others, hinders the transmission of information, the promotion of farewells and family support.” I2

According to Hanna et al. [21], the management and transmission of information about the patient may have been underestimated by health professionals and influenced by lack of communication skills or lack of preparation of professionals to transmit this information, in the final stage of life. It is important to understand what are the best practices around end-of-life communication during a pandemic.

Participants noted that there is a lack of specific policies, standards, and procedures.

Evidence-based guidelines for action may support clinical practice, allowing for a safe and adequate response.

The main contact of patients is with health professionals. Throughout the pandemic, physical touch such as a touch from a hand was replaced by the touch of gloves.

“The use of surgical masks and other personal protective equipment constitute a barrier to non-verbal communication, preventing, for example, touching, which alters the recipients perception of communication.” I5

Facial expression is distorted by the reflections of glasses and visors. The pandemic instigated anxiety and strain on health professionals. The fear of becoming infected and infecting other people prevails, causing a feeling of impotence [23].

PC includes, but is not limited to, end-of-life care. Palliative actions must be integrated into the fight against the disease. They should be practiced by all healthcare professionals who care for people infected with the new coronavirus. They include the relief of dyspnea and other symptoms and include psycho-emotional support for the patient and the family. Participants report difficulties in access to PC by patients, especially because, from their perspective, the treatment of acutely ill people is prioritized, to the detriment of people with acute chronic illness. This was reported by health professionals, to be an added difficulty in the symptomatic control of patients with palliative needs.

“I also believe that the pandemic influences our palliative patients' decision-making regarding access to healthcare services - the fear of infection, the balance between harm and benefit from the exposure. As a result, many patients come to us with a more established lack of symptomatic control and their management becomes more challenging.” I7

Screening patients with palliative needs in the respiratory circuit implies a longer time to establishing symptomatic control, creating barriers to access and increasing costs for health systems [4,16].

“When we triage in the emergency department and acute respiratory diseases gain priority over long-term chronic diseases, we are limiting our patients' access to PC units and, consequently, compromising symptomatic control.” I11

It is important to address the emotional impact on health professionals.

“Difficulties in providing care begin when colleagues are mobilized to be in the ‘front line’ - or in the ‘COVID wards’ (...).” I4

The work overload, with the implementation of new tasks and the reallocation of professionals to services dedicated to COVID-19, the fear of infecting their significant others, and the stress caused by constant changes in work dynamics make it difficult to provide PC.

Many hope that the pandemic will promote a better assessment of the critical nature of PC in order to respond more efficiently to suffering. It is urgent to recognize that PC is an essential and important part of healthcare, equal to and, in some situations, more appropriate than intensive care. Improving access to PC has never been more important. 

Strategies to be implemented from the perspective of health professionals 

Exceptional times call for exceptional measures. Over the past few months, healthcare professionals and healthcare institutions have been developing their search for solutions and alternatives, for patients with and without COVID-19. The healthcare response to the current challenge has evolved with growing experience and knowledge of the ongoing pandemic. Analysis of the experience accumulated to date allows responses to be adapted as needed.

The ultimate safety of the patient constitutes one of the great challenges for healthcare in the 21st century, so it is essential to recognize errors or lapses of care, which can have serious consequences for patients and their families and health institutions. Communication is, according to the reports of health professionals, a decisive factor of quality and safety in the provision of care, so the identification of problems and strategies that promote effective communication is essential to reduce the impact of the COVID-19 pandemic.

One of the strategies mentioned that reduce the impact caused by visit restrictions is programmed telephone contacts and video calls, minimizing the decrease in physical contact caused by visit restrictions.

“(...) telephone contacts for family members in order to keep them well informed and secure about the care being provided to the patient.” I5

Early establishment of who is the family member or person of reference for the transmission of information related to the hospitalized patient is recommended.

The family, vital in supporting the palliative patient, experiences, alongside the patient, some type of suffering. At a time of heightened vulnerability, it is important to involve the patient´s family and their significant others [21]. Health professionals report that family conferences, as a structured form of intervention in the family can be helpful. They should respect previously defined objectives, allow for clarification and sharing of clinical information, share feelings between the different participants present, and, if necessary, help to target patterns of interaction in the family, possibly improving the relationship between the patient and their family [24]. Communication is one of the pillars of PC, along with symptom control, family support, and teamwork. Adequate communication works as an intervention strategy in suffering and control of symptoms associated with an advanced and terminal illness. Efficacy in communication is sought and for this, it is imperative that there is training. Both training and experience of health professionals have been reported in this study as essential to reduce the impact of the COVID-19 pandemic in the provision of PC.

Another strategy described by health professionals is the possibility of having a family member or significant other, present 24 hours a day, by the patient´s side, during the last days and hours of life, in order to allow for goodbyes and mourning.

“(...) the patient was in the last days/hours of life and 1/2 family members were allowed to be present for a short period of time (always complying with the norms of the General Health Department) so that they could say goodbye and the patient could fulfill some end-of-life tasks.” I10

They also add that another strategy that can help to reduce negative emotions felt by the patient and family members is permission for a postmortem visit.

“(...) possibility of 1 or 2 family members being able to travel to the unit after the death of the loved one to see, touch, pray and say goodbye (since funeral ceremonies were limited with no possibility of an open casket). The importance of the family being able to see the deceased can be crucial to the grieving process.” I10

The creation and update of norms related to practices and procedures adapted to the epidemiological context, promotes, according to health professionals, a reduction in the impact of the pandemic on the provision of care.

The implementation of measures that promote the well-being of health professionals is important. Measures to identify stress-inducing factors, redefine working hours to allow for periods of adequate rest, and effective multidisciplinary teamwork, which is consistent with recent literature [6].

“Teamwork and a present and empathetic leader, who is clear and calm regarding their leadership strategies, also evokes security and improves commitment and cohesion among health professionals.” I12

Mutual respect, trust, and closeness between different professionals in the team helped to recognize signs of distress, burnout and concerns about mental health.

Study limitations

The data collection phase took place between the fifth and sixth months after the declaration of the pandemic, so the difficulties reflect the limited knowledge at the time.

The study sample refers only to a tertiary-level PC unit that attends to complex patients. The time available for carrying out the study may have influenced the results since the participation of health professionals from other teams dedicated to providing PC could have been obtained.

The sample, although multidisciplinary, does not reflect the entire PC team of the PCU under study, since other professional groups are also part of it, but it did not meet the inclusion criteria. Given the nature of the study and the data, no statistical analysis was carried out.

The use of focus groups for data collection, as a form of group interview, with the aim of exploring the dynamics within a team, constitutes a valuable method of data collection in qualitative research. Since it was not possible to form representative groups, given the impossibility of gathering the sample, we opted for the use of individual written narratives and, in this way, the data obtained reflects an individual reflection.

## Conclusions

Isolation from the family and social circle, the perception of risk for the entire family due to the transmission of infection, with the added uncertainty about its effect and, in some cases, the guilt of having been a possible transmitter, are factors that contribute to the emotional response to this pandemic. The reduction in contact between patients and health professionals, knowing that proximity and physical contact can be therapeutic, is also a factor that contributes to the increase in suffering. The use of personal protective equipment and physical distance are barriers to communication and emotional support, which becomes even more necessary in the absence of the family and significant others.

Health professionals and caregivers face enormous pressure, but also difficulty in providing individualized support. Many feel overwhelmed by current circumstances and in need of support. Resilience among health professionals should be promoted through support measures and teamwork.

PC must be provided to all who need it, with or without COVID-19. During this pandemic, as well as limiting the spread of disease and increasing chances of survival, we must also aim to improve quality of life, provide comfort, dignity, and compassion to those who so desperately need it.

## References

[1] (2020). World Health Organisation (WHO). Coronavirus disease. https://www.who.int/publications/m/item/situation-report---51.

[2] (2020). Instituto Nacional de Estatística - INE (Statistics Portugal). Mortality in Portugal in the context of the Covid-19 pandemic. https://www.ine.pt/xportal/xmain?xpid=INE&xpgid=ine_destaques&DESTAQUESdest_boui=452172550&DESTAQUESmodo=2&xlang=pt..

[3] (2020). Entidade Reguladora da Saúde - ERS (Portuguese Health Regulatory Authority). Monitoring information - Impact of the COVID-19 pandemic on the Health System - period from March to June 2020. https://www.ers.pt/media/3487/im-impacto-covid-19.pdf.

[4] Arya A, Buchman S, Gagnon B, Downar J (2020). Pandemic palliative care: beyond ventilators and saving lives. CMAJ.

[5] Chapman M, Russell B, Philip J (2020). Systems of care in crisis: The changing nature of palliative care during COVID-19. J Bioeth Inq.

[6] Pastrana T, De Lima L, Pettus K, Ramsey A, Napier G, Wenk R, Radbruch L (2021). The impact of COVID-19 on palliative care workers across the world: a qualitative analysis of responses to open-ended questions. Palliat Support Care.

[7] Oluyase A, Hocaoglu M, Cripps R (2020). The challenges of caring for people dying from COVID- 19: a multinational, observational study of palliative and hospice services (CovPall) [PREPRINT]. Cold Spring Harbor Laboratory.

[8] Radbruch L, Payne S (2010). White paper on standards and norms for hospice and palliative care in Europe: part 2. Eur J Palliative Care.

[9] Twycross R (2020). Palliative care: what, who, when, how?. World Med J.

[10] Chochinov HM, Bolton J, Sareen J (2020). Death, dying, and dignity in the time of the COVID-19 pandemic. J Palliat Med.

[11] (2019). Observatório Nacional dos Cuidados Paliativos - OPCP (Portuguese Palliative Care Observatory). Fall 2019 Report - Coverage and Characterization of Palliative Care Teams and Professionals. https://ics.lisboa.ucp.pt/asset/4181/file.

[12] (2019). Observatório Nacional dos Cuidados Paliativos - OPCP (Portuguese Palliative Care Observatory). Fall 2019 Report - Assistance Activity of Palliative Care Teams/Services. https://ics.lisboa.ucp.pt/asset/4221/file.

[13] Abbott J, Johnson D, Wynia M (2020). Ensuring adequate palliative and hospice care during COVID-19 surges. JAMA.

[14] Fadul N, Elsayem AF, Bruera E (2021). Integration of palliative care into COVID-19 pandemic planning. BMJ Support Palliat Care.

[15] Knights D, Knights F, Lawrie I (2020). Upside down solutions: palliative care and COVID-19 [PREPRINT]. BMJ Support Palliat Care.

[16] Byock IR (1996). The nature of suffering and the nature of opportunity at the end of life. Clinics Geriatric Med.

[17] Bardin L (2011). Content analysis, 4th ed.. 4th ed. Edições 70.

[18] Chochinov HM (2007). Dignity and the essence of medicine: the A, B, C, and D of dignity conserving care. BMJ.

[19] Abad C, Fearday A, Safdar N (2010). Adverse effects of isolation in hospitalised patients: a systematic review. J Hosp Infect.

[20] Marra A, Buonanno P, Vargas M, Iacovazzo C, Ely EW, Servillo G (2020). How COVID-19 pandemic changed our communication with families: losing nonverbal cues. Crit Care.

[21] Hanna JR, Rapa E, Dalton LJ (2021). A qualitative study of bereaved relatives' end of life experiences during the COVID-19 pandemic. Palliat Med.

[22] Back A, Tulsky JA, Arnold RM (2020). Communication skills in the age of COVID-19. Ann Intern Med.

[23] Carvalheiro AM, Faria C, Semeão I, Martinho SM (2021). Caring for end-of-life patients and their families, during life, and mourning, in the COVID-19 era-the experience of a palliative care team in Portugal. Front Psychiatry.

[24] Neto I (2003). The family conference as an instrument to support the family in palliative care. Revista Portuguesa de Medicina Geral e Familiar.

